# Micro-blog user community discovery using generalized SimRank edge weighting method

**DOI:** 10.1371/journal.pone.0196447

**Published:** 2018-05-07

**Authors:** Jinshan Qi, Liang Xun, Xiaoping Zhou, Zhiyu Li, Yu Liu, Hengchao Cheng

**Affiliations:** 1 School of Information, Renmin University of China, Beijing, China; 2 School of Computer Science and Technology, Huaiyin Normal University, Huaian, China; Universidad Nacional de Mar del Plata, ARGENTINA

## Abstract

Community discovery is one of the most popular issues in analyzing and understanding a network. Previous research suggests that the discovery can be enhanced by assigning weights to the edges of the network. This paper proposes a novel edge weighting method, which balances both local and global weighting based on the idea of shared neighbor ranging between users and the interpersonal significance of the social network community. We assume that users belonging to the same community have similar relationship network structures. By controlling the measure of “neighborhood”, this method can adequately adapt to real-world networks. Therefore, the famous similarity calculation method—SimRank—can be regarded as a special case of our method. According to the practical significance of social networks, we propose a new evaluation method that uses the communication rate to measure its divided demerit to better express users’ interaction relations than the ordinary modularity *Q*. Furthermore, the fast Newman algorithm is extended to weighted networks. In addition, we use four real networks in the largest Chinese micro-blog website Sina. The results of experiments demonstrate that the proposed method easily meets the balancing requirements and is more robust to different kinds of networks. The experimental results also indicate that the proposed algorithm outperforms several conventional weighting methods.

## Introduction

Networks (or graphs) such as collaboration networks and social networks have been proved to be ubiquitous and effective as natural models for many real-world systems of interacting entities. The well-established branch of graph analysis methods in mathematics and computer science enables us to analyze social networks more efficiently.

In a network, a community is informally defined as an “unusually densely connected sets of nodes (or vertices)”. Community discovery is a common interest in analyzing and understanding such network systems for its several benefits [1]. However, determining how to identify the community structure of a given network remains a very difficult problem, and large amounts of work have been done [2–5]. Two categories of existing methods can be adopted to identify the community structure in a network. The first category consists of non-overlapping community discovery algorithms. They divide the complex network into several non-connected communities, such that every node belongs to one particular community. More specifically, this category of algorithms includes the hierarchical clustering method, spectral clustering method and modularity optimization method. The hierarchical clustering method first defines the similarity of or distance between nodes in the network according to the network topology structure. Then, it organizes the nodes into a tree hierarchy using single-connection hierarchical clustering or full-connection hierarchical clustering. By crosscutting the tree in different ways, different community division results are obtained to meet different needs. The method in [6] is a representative one. The spectral clustering method comes from the graph partitioning problem. To solve this problem, one must find a method to cut the fewest edges to obtain a disjoint set of nodes. A typical algorithm is given in [7], and a similar one is presented in [8]. The idea of the modularity optimization method is that the inter-community conjunctive probability should be higher than that of a random sequence with the same degree. Its evaluation function is the modularity *Q*. A higher value of *Q* demonstrates better structure of the community. The simulated annealing algorithm is a typical implementation of this method [9]. The second category of algorithms consists of the overlapping community discovery algorithms, which allow each node to belong to multiple communities at the same time. In other words, there is overlap between communities. The Community Overlap PRopagation Algorithm(COPRA) in [10] is a representative algorithm, which discovers the overlapping communities by the label propagation algorithm. In the initial stage, every node is assigned as a unique label. Then, the label as well as its degree of membership are updated by iteration. At last, the nodes that have the same label are classified into the same community. If a node has multiple labels, it connects different communities. The Constrained Label Propagation Algorithm(CLPA) algorithm detects the main communities in a network by using the number of mutual neighboring nodes, and then nodes are added into communities by using a constrained LPA [11]. The Link Clustering(LC) algorithm first clusters the edges and obtains the edge communities, and then transforms the edge communities into corresponding node communities. The nodes belonging to several communities are the overlapped nodes [12]. Some similar algorithms are presented in [13–15]. There are algorithms based on local community optimization and expansion; for example, the Lancichinetti Fortunato Method(LFM) starts from a seed user that was chosen randomly and performs outward expansion to build communities until the community function attains a local optimum [16]. The MaxPerm algorithm detects communities by maximizing the permanence of the network [17]. Reference [18] presents a local expansion method by density-based clustering, which aims to uncover the intrinsic network communities by locating the structural centers of communities based on a proposed structural centrality. Topological potential is another novel theory of overlapping community discovery, which has been extended by some other relevant algorithms. Article [19] provides an overlapping community discovery method that depends on the analysis of locations of nodes, which uses PageRank to evaluate the quality of each node and determine the community relationships according to the node locations in the inherent peak and valley structure of the topological potential. A multi-objective approach named MOGA-MDNet discovers communities in multidimensional networks by applying genetic algorithms [20].

The idea of edge weighting for community discovery algorithms was initially introduced by M. Girvan and M. E. J. Newman in the Girvan-Newman Algorithm [21], where they weighted every edge of the network by using the modified edge betweenness and removed the “most between edges” repeatedly. As a result, the network can be divided into small groups gradually. This idea proved to be effective, but unfortunately limited by the drawback of prohibitively large computational cost, as it needs to recalculate the weights of edges in each step. Several faster algorithms have been proposed in later literature, such as the fast Newman algorithm [22] and the Clauset-Newman-Moore method (CNM) [23]. However, these methods fail to take edge weighting into consideration, and their performances were sometimes reported to be poor. Therefore, a natural idea is to combine the edge weighting methods with these fast algorithms to balance the performance and computational complexity [24–26]. Conventional edge weighting methods include the inverse edge betweenness, edge clustering coefficient, and common neighbor ratio.

When applying an edge weighting method, one needs to think about whether the local or global characteristics should be considered in the method. A global weighting method must collect information on the global scale of the network for the weight computation of a single edge. It seems unimpeachable to consider the structure of the entire network for edge weighting. However, this could lead to some problems. First, it is apparent that a heavier computational cost is incurred. Second, this approach might deteriorate the performance of the original community discovery algorithm when the local community structure is important, because this kind of local information is easily covered by the excess global information. In contrast, local weighting methods consider more local information, which could make them more efficient. However, these methods face the risk of missing information on a larger scale. Therefore, to obtain an efficient and effective edge weighting method, it is important to balance the local and global weightings. Although many edge weighting schemes have been developed and successfully applied to cope with the community discovery problem, most are not well balanced. S. Asur et al. [24] combine the clustering coefficient and betweenness, which weights the graph, to balance the local and global weightings. A. Khadiviet et al. [25] developed an edge weighting scheme by integrating the edge betweenness and the common neighbor ratio.

Micro-blog is an information sharing and dissemination platform based on users’ creation and following of micro-blogging posts. Users can post micro-blogs of less than 140 characters using their website clients, mobile phone clients or third-party applications, anywhere and at any time. According to the China Internet Network Information Center (CNNIC) in January 2013, data show that at the end of December 2012, there were 309 million micro-blogging users in China. Compared to the end of 2011, there was an increase of 58.73 million users. The annual growth rate reached 23.5%. Sina.com launched its micro-blog service in August 2009, and has the largest community of micro-blog users in China. Currently, its data size is over 1 PB. It is becoming the mainstream platform for people to access information and exchange experiences.

Micro-blogs allow users to subscribe to messages from anyone without permission so that users can follow anyone they like, get information from their followers, and post comments to their followers. These following relationships build dominant or recessive communities that reflect users’ common interests, similar experiences or awareness of a certain topic. The micro-blog social network has the following characterizes. First, as a social network, it has the Six Degrees of Separation characteristic: human society is a small-world-type network characterized by short path-lengths, and a person is connected to anyone in the world through less than six people [27]. People are becoming more connected than ever before. Second, a network in a micro-blog is very complex and confusing because we can follow anyone we like without their permission. This allows us to make friends with people from different backgrounds and various places, thereby building an obstacle to division of the community. Third, a micro-blog contains an enormous amount of data and is very difficult to capture and store. Identifying communities among ultra-large-scale micro-blog users is a very important task, and has many practical implications with a wide range of information. Community discovery on the micro-blog platform can reveal the relationships more clearly. The obtained communities can help manufacturers and vendors find potential buyers for their products more accurately for precision marketing. They can also guide users to find the most interesting information. Community structure can also be used to analyze the generation and propagation mechanisms of topic evolution in a micro-blog network.

The remainder of this paper is organized as follows. We present the related research about edge weighting methods for community detection in Section 2. Section 3 provides a detailed discussion of the edge weighting methods. The algorithm proposed in this study is presented in Section 4. In Section 5, experimental studies are employed to demonstrate the performance of the new weighting scheme and compared with other weighting methods. Finally, conclusions and future research directions are summarized in Section 6.

## Related work

The existing works related to the present study are introduced in this section. The GN and fast Newman algorithms, the SimRank edge weighting algorithm, and the concept of modularity are discussed.

### GN and fast Newman algorithms

In recent years, many community discovery algorithms have been proposed. Among these methods, the GN algorithm [21] and its derivatives are especially striking. The motivation behind this algorithm is that the edges between communities can be thought of as bottlenecks of communication between communities. The GN algorithm uses edge betweenness to measure the flow of edges to weight and remove edges. The GN algorithm was proved to be effective in most cases, but unfortunately is limited by high computational complexity: O(m^2^n) or O(n^3^) for sparse graphs, where m and n represent the numbers of edges and nodes, respectively. To improve the GN algorithm, Tyler et al. [28] have proposed an algorithm that only selects a subset of the nodes as source nodes. Radicchi et al. [5] have proposed a self-contained GN algorithm that uses a measure different from betweenness.

To reduce the computational complexity, the other two techniques based on optimizing the modularity in a greedy way were developed afterwards: the fast Newman algorithm [22] and CNM [23]. In these two methods, every single node is treated as a community in the beginning, and the two communities that bring about the largest increase (or smallest decrease) in ΔQ… are merged in each step. For sparse graphs, the computational complexity of the fast Newman algorithm is O(*m*+*n*)*n*) or O(*n*^2^), while it is O(*md*log(*n*)) or O(*n*log^2^(*n*)) for CNM, where *d* indicates the depth of the dendrogram. The time savings of these two methods were significant, but the performance on community identification was sometimes reported to be poor. The problem lies in the fact that all the edges are treated the same, which leads to the failure to make full use of the information that the network carries.

### Edge weighting scheme

Existing works show that the detection of communities can be enhanced by proper weighting of edges in a network. There are a variety of ways to weight the edges of a given network, such as using edge betweennesses, edge clustering coefficients and common neighbor similarities or ratios. The betweenness of an edge is defined as the fraction of shortest paths between any pair of nodes that run along this edge. In most cases, one can expect that it provides an efficient metric to distinguish among edges between communities and edges within a community. The edge betweenness *B*_*ij*_ of the edge between nodes *i* and *j* is expressed as Eq (1),

$$B_{ij}=\sum_{u\neq v\in V}\frac{\sigma (e_{ij})}{\sigma_{uv}}$$ (1)

where *σ*(*e*_*ij*_) is the number of shortest paths between nodes *u* and *v* that pass through edge *e*_*ij*_ and *σ*_*uv*_ is the total number of shortest paths between *u* and *v*.

Another commonly used similarity measure is the edge clustering coefficient proposed by Radicchi et al. [5]. The edge clustering coefficient is based on counting triangles of edges in the network. Consider an edge that runs between node *i* and node *j*, whose degrees are *k*_*i*_ and *k*_*j*_. The maximum number of triangles containing this edge is min{*k*_*i*_—1, *k*_*j*_—1} and the actual number is *z*_*ij*_. Then, the edge clustering coefficient *C*_*ij*,_ which represents the fraction of actual triangles, is expressed as Eq (2),

$$C_{ij}=\frac{z_{ij}+1}{\text{min}\{k_{i}-1,k_{j}-1\}}$$ (2)

Radicchi et al. showed that the edge clustering coefficient is strongly negatively correlated with the edge betweenness in the network in which they appear. This means an edge for which the edge clustering coefficient is small may run between communities.

The common neighbor ratio can also be used to measure the similarity between nodes and, thus, to weight edges. To compute the weight of edge *e*(*i*, *j*) by using the common neighbor ratio, the number of shared neighbors and the total numbers of neighbors of nodes *i* and *j* are first determined. Then, the weight of edge *e*(*i*, *j*) is calculated,

$$w(i,j)=\frac{\left|\text{neighbor}(i)\cap \text{neighbor}(j)\right|}{\left|\text{neighbor}(i)\cup \text{neighbor}(j)\right|}$$ (3)

where neighbor(*i*) and neighbor(*j*) denote the neighbor sets of nodes *i* and *j*, respectively. The larger *w*(*i*, *j*) is, the more similar these two nodes are.

In the literature, there have been some discussions regarding the hybrid approaches for similarity measurement. For instance, S. Asur et al. [24] combined the clustering coefficient and betweenness to weight the graph. A. Khadivi et al. [25] used two well-known structural measures of complex networks: the edge betweenness centrality and common neighbor ratio. W. Berry et al. [26] proposed a weighting method based on the definition of a module [29].

### SimRank neighbor ranging algorithm

As mentioned in the previous section, the common neighbor ratio (CNR) is a well-known edge weighting method. The basic idea of CNR is that two nodes are more similar if they share more neighbors. Since the computation of similarity for the adjacent nodes is limited only to the relationship among the neighbors, it is a local weighting method. To enhance the performance of CNR, SimRank is introduced to extend the definition of CNR. The fundamental method of SimRank is similar to that of CNR in the idea that “two objects are similar if they are related to similar objects”.

Let *s* (*a*, *b*) ∈ [0, 1] denote the similarity between nodes *a* and *b*. If *a* = *b*, then *s* (*a*, *b*) = 1; otherwise,

$$s(a,b)=\frac{\gamma }{\left|N(a)||N(b)\right|}\sum_{i=1}^{\left|N(a)\right|}\sum_{j=1}^{\left|N(b)\right|}s(N_{i}(a),N_{j}(b))$$ (4)

where *γ* is a constant between 0 and 1, *N* (•) denotes the set consisting of node •’s neighbors, and |•| represents the cardinality of set •. If *N*(*a*) = *Φ* or *N*(*b*) = *Φ*, then *s*(*a*, *b*) = 0. However, this would never occur for an undirected connected graph.

The original SimRank computes the similarity of any pair of nodes in an iterative way until it converges. Initially,

$$s_{0}(a,b)=\left\{ \begin{array}{l} 0,\;\text{if}\;a\neq b\\ 1,\;\text{if}\;a=b \end{array}\right.$$ (5)

Then, in iteration *k*, we compute *s*_*k*_ (*a*, *b*) from *s*_*k*-1_(*, *),

$$s_{k}(a,b)=\frac{\gamma }{\left|N(a)||N(b)\right|}\sum_{i=1}^{\left|N(a)\right|}\sum_{j=1}^{\left|N(b)\right|}s_{k-1}(N_{i}(a),N_{j}(b))$$ (6)

for *a* ≠ *b*, and *s*_*k*_(*a*, *b*) = 1 for *a* = *b*. It has been proved in the original work that the similarity of node pairs will always converge in a reasonable number of iterations *K*; usually, *K* = 5.

The computing procedure of SimRank reveals that it is actually a generalization of the previous approaches that compute similarity by common neighbors alone. At the very beginning, in the first iteration, the algorithm calculates the similarity of each node pair by using only the information of their direct neighbors. With the increase of the iteration number, the node pairs gradually get more information away from themselves for the similarity computation. Finally, the similarity converges as the information of the whole network is collected. In other words, SimRank can be treated as a weighting method that is local in the beginning and then globalized gradually during the procedure.

## Problem formulations

As discussed in the previous section, a number of edge weighting schemes have been proposed. These weighting schemes are generally implemented in two ways. The first way is directly based on the attributes of edges, such as the SimRank neighbor ratio. The second way is based on the similarity or distance of the node pair that was employed to measure the weight of the corresponding edge; the common neighbor ratio and clustering coefficient, for instance, fall into this category. It must be noted here that although the clustering coefficient seems to analyze the number of triangles in which a given edge is involved, it is essential to study the common neighbors, which is extremely similar to calculating the common neighbor ratio. Meanwhile, according to the scope of the network involved in the computing procedure, these weighting schemes can also be divided into two categories: the local weighting scheme and the global weighting scheme. These two categories of methods have different characteristics. An obvious disparity is in the computational complexity. Moreover, the computation of the weight for a given edge with a local weighting method is usually based only on the local information of the network. In contrast, a global weighting scheme collects the information on the global scale for the weight computing procedure of any single edge. Analyzing the corresponding computation procedure, the SimRank neighbor ratio can be categorized as a global weighting scheme, while the common neighbor ratio and clustering coefficient are local weighting methods.

**Fig 1:**
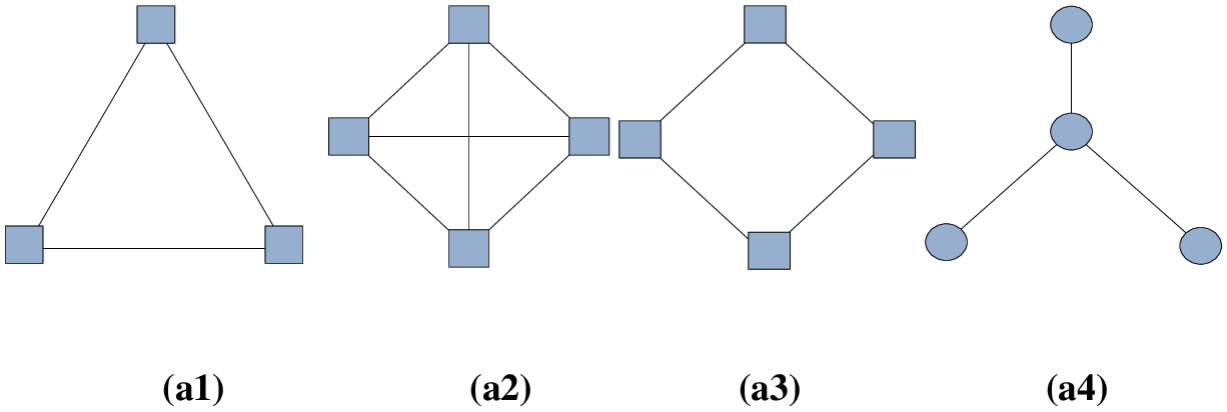
Examples of local weighting schemes: (a1) clique with 3 nodes, (a2) clique with 4 nodes, (a3), (a4) graphs with no triangles. In other words, no two nodes have common neighbors.

The common neighbor ratio measures the fraction of neighbors that two nodes share. In fact, the common neighbor number is equal to the number of triangles in which the corresponding edge is involved. It is not difficult to obtain the relationship shown as Eq (7).

$$z_{ij}=|\text{neighbor}(i)\cap \text{neighbor}(j)|$$ (7)

where *z*_*ij*_ denotes the number of triangles that contain the edge and neighbor(*k*) represents the set of neighbors of node *k*. It has to be noted that triangles are the most common *clique* in a network (ignoring single nodes and node pairs). A *clique* refers to a complete sub-graph in a network. In this CNR method, only nodes that share a neighbor can be defined as friends. It only takes close connections in consideration; therefore, it is a local weighting scheme. For instance, Fig 1(a1) and 1(a2) depict cliques with 3 and 4 nodes, respectively. The common neighbor ratio or edge clustering coefficient are effective for these kinds of network structures, where the connections between nodes are relatively close. In contrast, for some sparse graphs, these two local weighting methods may not be able to collect enough information for a good decomposition. Fig 1(a3) and 1(a4) show two graphs with no triangles, that is to say, no two nodes have common neighbors; thus, the weights of all edges would be 0 if either of these two weighting schemes were employed.

According to the aforementioned analysis, the SimRank neighbor ratio first considers the information of the direct neighbors. However, with the increase of the iteration number *K*, information of nodes that are farther away from the node pair is gradually considered, and information of the whole network is considered in the calculation when the iteration number is large enough. This means that a larger *K* value indicates that more global information is collected. The common choice of *K* is 5 in SimRank, which means a large amount of distant information is considered. Obviously, the SimRank neighbor ratio is a global weighting method. For example, Fig 1(a3) and 1(a4) show that graphs with no triangles can use the SimRank neighbor ratio to calculate their similarity because this method uses more distant information.

**Fig 2:**
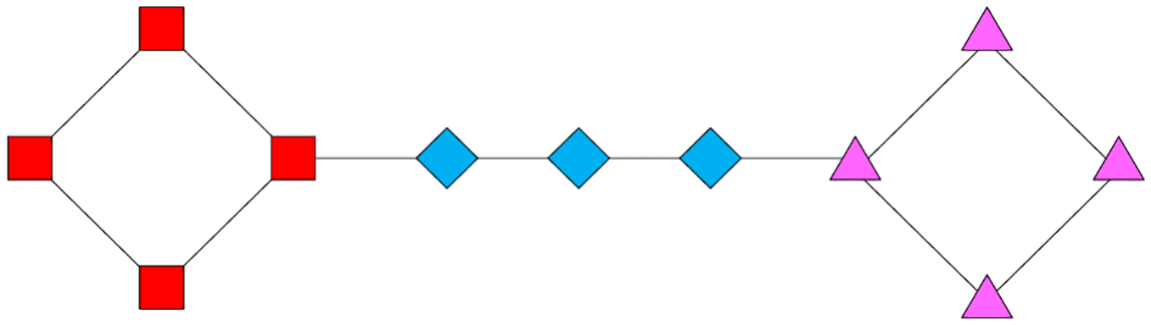
A network with 11 nodes. Different colors represent different clusters.

However, in a real social network, the size of the network is very large and the network contains a large number of user nodes. It can be seen from Fig 1 that a network with 3 or 4 nodes cannot represent the inner structure of the social network. Fig 2 shows a larger graph, which can be divided into three communities visually. It is obvious that the local weighting methods cannot be used to assign proper weights for edges because they treat all the edges equally. In contrast, the SimRank neighbor ratio, which takes more distant information into consideration, can be used in this kind of network. However, the SimRank neighbor ratio usually considers an excessive amount of external information, so the network community may not be precisely divided. In Fig 2, after a few iterations, nodes in different communities gradually increase in similarity, eventually leading to all 11 nodes being divided into only one community. This is because the SimRank neighbor ratio does not have any restrictions in considering the relationships in large network structures and the distance relationship is still considered when calculating the similarity of node pairs.

The discussion reveals the problem that the structure of a given network should be taken into consideration when weighting edges. In some cases, more attention should be paid to the global information when dealing with very sparse networks. In some other cases, the local information for a single community is more valuable, such as for networks with extremely unbalanced community scales. Obviously, an algorithm can consider a certain limitation of the scope of the information and node similarity. The algorithm proposed in Section 4 can reach a good balance between local and global weighting and adapt to real-world social networks.

## Materials and methods

In this section, a generalized SimRank neighbor weighting method is proposed that can balance the local and global weighting and adapt to the practical significance of the social network. Next, the fast Newman algorithm is extended to weighted networks. The proposed edge weighting scheme enhances the performance of the fast Newman algorithm significantly. Last, we propose a new evaluation method: using the communication rate to measure its divided demerit, users’ interaction relations can be better expressed than with the ordinary modularity measure *Q*.

### The generalized SimRank neighbor weighting scheme

**Fig 3:**
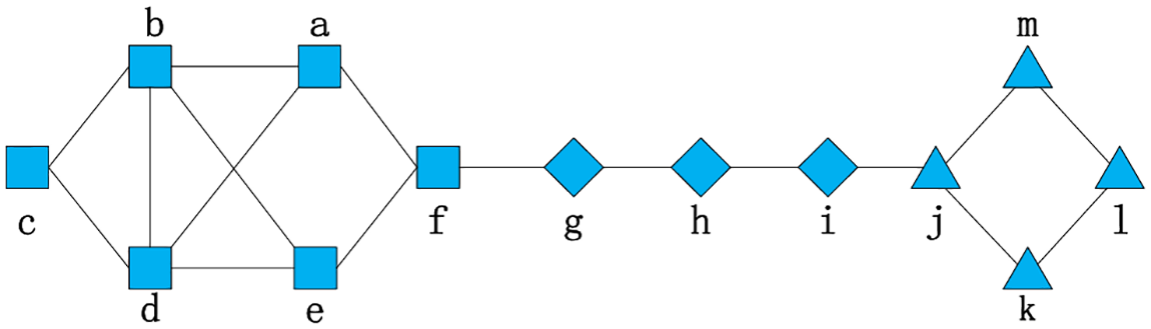
A reduced example of a real social network.

As mentioned in Section 2, SimRank calculates the similarity between users based on their common friends and, finally, takes the information of the whole network into consideration. This has strong practical significance in a social network: a pair of users are similar if they are related to the same user [30]. However, this algorithm has some drawbacks when applied to a real-world social network. We propose our novel neighbor weighting scheme to improve these shortcomings. In Fig 3, it is assumed that the network is an example of a real social network with 12 nodes. Each node represents a real user, identified by a letter, and the line connecting nodes indicates there is a following relationship between the corresponding pair of users. We will explain our improved methods with the help of this figure.

#### The advanced initiation method

In the initialization of the original SimRank algorithm, *s* (*a*, *b*) = 1 if *a* = *b*, and otherwise, *s* (*a*, *b*) = 0; here, *a* and *b* denote nodes in a network. In other words, the input of the original SimRank algorithm is a diagonal matrix. This means in the 0th iteration, the similarity of *a* and *b* will be 0, regardless of whether they are “friends” or not. However, if there are few friends between *a* and *b*, their similarity will always be 0 in earlier iterations and, in some cases, two of the friend points may share a similarity of 0 in the end. In Fig 3, we can find that *k* treats *j* and *l* as its friends, and *l* has a friend list that contains *k* and *m*. At first, *s* (*k*, *l*) = 0; then, in the following iterations, *S*_*k*-1_(*k*, *l*) will be added to *S*_*k*_(*k*, *l*) based on Eq (6) in Section 2.3. However, this is a very slow process and the similarity between *k* and *l* is close to zero in the end. This leads us to think that we should use discriminatory analysis to compare different relations between *a* and *b*.

In our work, we change the initialization of SimRank to ensure that any pair of nodes that has a directed connection will have a positive similarity. In Eq (8), we define the similarity between nodes *a* and *b* using their degrees if they have a direct connection in the network. Here, *I*(*a*) refers to the degree of node *a*. The symbol *a* ^*b* means users *a* and *b* have a direct connection.

R0(a,b)={1if(a=b)1I(a)*I(b)if(a^b)0else (8)

We contrast the performances obtained using this advanced initialization method and the original method in Table 1. Sim(*k*, *l*) refers to the similarity between nodes *l* and *k* in Fig 3. Sim(*k*, *l*) with the original SimRank initialization is 0 until the 9th iteration, but with the advanced initialization, Sim(*k*, *l*) eventually becomes stable at 0.1060, which is much larger than Sim(*k*, *l*) with the original initialization.

**Table 1:** Similarity between *k* and *l* in the original initialization and advanced initialization.

Iteration *K*	Sim(*k*, *l*) in the original initialization	Sim(*k*, *l*) in the advanced initialization
0	0	0.25
3	0	0.1736
6	0	0.1477
9	0.0001	0.1308
12	0.0008	0.1182
15	0.0035	0.1098
18	0.0093	0.1060
20	0.0150	0.1060

#### The novel neighbor weighting method

SimRank calculates the similarity between users based on their common friends and, finally, takes the information of the whole network into consideration. In this case, in the original SimRank, the similarity between two users, as similarity between indirect friends is passed into the network, may reach a relatively large value after a certain number of iterations. However, there will be a redundancy in community discovery if we incorporate all these connections into the computation since the real social network has the characteristic of six degrees of separation. This is the main drawback of the SimRank algorithms. Based on this, we propose a **Restricted Neighbor Ranging Method** (**RNRM**) based on the SimRank to exclude the redundant information. Only if a pair of users has one or more pairs of common friends will their common friends’ similarities be in used in future iterations. The new similarity method is shown in Eq (9).

$$S_{k}(a,b)=\frac{\gamma }{\left|I(a)||I(b)\right|}\sum_{i=1}^{Z(a)}\sum_{j=1}^{Z(b)}S_{k-1}(Z_{i}(a),Z_{j}(b))$$ (9)

where *γ* is a constant between 0 and 1. We put the common neighbors between *a* and *b* into *Z*(*a*) and *Z*(*b*). To ensure that whether *a* and *b* are connected is reflected in the equation, we put *b* into *Z*(*a*) and *a* into *Z*(*b*) if *a* and *b* are connected. This means that if *a* and *b* are part of a triangle, the similarities of the other two sides will contribute to the similarity of *a* and *b*. Using this method, we can avoid the impact of the small-world characteristic.

Here, we still consider the network in Fig 3 as an example. Users *f* and *j* have no direct relation and *f* can be introduced to *j* by a list of friends through *g→h→i*. Obviously, in our daily lives, users like this actually have an alienated relationship and barely have a chance to get to know each other, so the similarity of users *f* and *j* should be low. However, when using the original SimRank, their similarity in the iterative process depends on the similarities of nine user pairs, such as *g* & *i*, *e* & *i*, and *a* & *i*. This causes an improvement in their similarity. In our algorithm, only if a pair of users are both common friends of *f* and *j* will their similarity will be used in the computation. Since there is no friend in Fig 3 that satisfies this criterion, Sim(*f*, *j*) is 0 from beginning to end. Table 2 shows the similarity between user *f* and user *j*, calculated using the original SimRank and the Restricted Neighbor Ranging Method.

**Table 2:** Similarity between *f* and *j* in original SimRank and RNRM.

Iteration *K*	Sim(*f*, *j*) in the SimRank with original initialization	Sim(*f*, *j*) in the RNRM
0	0	0
3	0.0525	0
6	0.1410	0
9	0.2102	0
12	0.2772	0
15	0.3206	0
18	0.3666	0
20	0.3936	0

In the original SimRank, the similarity between *f* and *j* is 0.3936 in the 20th iteration and is still increasing, but in our RNRM, the similarity remains 0 all the time. Thus, in the RNRM, two groups that have a connection such as “*f*-g-*h*-*i*-*j*” can be separated more easily.

**Fig 4:**
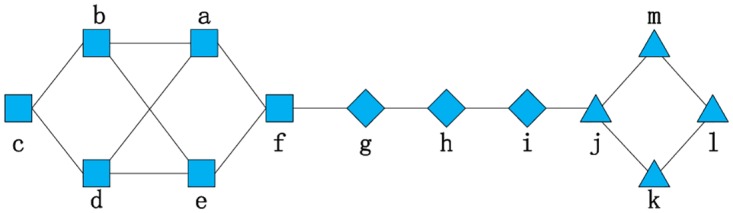
Users *b* and *d* don’t have a direct connection, in contrast to Fig 3.

In Fig 3, for users *a* and *e*, user *b* and user *d* are their common friends and themselves have a direct connection. However, in real life, *b* and *d* may not know each other. For comparison, we suppose that users *b* and *d* don’t have a direct connection in Fig 4.

When dealing with RNRM, the similarities between users *a* and *e* in Figs 3 and 4 are relatively equal since RNRM treats these two kinds of relationships between users *b* and *d* the same. However, we can easily find that there is still a difference between these two figures. In this case, a more rigorous criterion for the similarity used in the iteration is needed. We can specify that only when their common friends have a direct connection can their friends’ similarity be added to the similarity between users *a* and *b*. Therefore, in Fig 4, users *b* and *d* do not have an impact on the similarity between users *a* and *e* under this rigorous restriction. We called this the **Restricted Neighbor Ranging Method++ (RNRM++)**. The condition is defined as follows:

$$I_{i}(a)\in I(b)\cup b\;\text{and}\;I_{i}(b)\in I(a)\cup a\;\text{and}\;I_{i}(a)\cup I_{i}(b)$$ (10)

To evaluate our algorithm, we show the similarities between user *a* and user *e* based on Figs 3 and 4 calculated using RNRM++, and display Sim(*a*, *e*) based on Fig 4 calculated using RNRM in Table 3.

**Table 3:** Similarities between *a* and *e* calculated using RNRM++ and RNRM.

Iteration *K*	Sim(*a*, *e*) in Fig 3 using RNRM++	Sim(a, *e*) in Fig 4 using RNRM++	Sim(*a*, *e*) in Fig 4 using RNRM
0	0	0	0
2	0.3840	0.3333	0.5062
4	0.3905	0.3333	0.6251
6	0.3914	0.3333	0.6529
8	0.3915	0.3333	0.6594
10	0.3915	0.3333	0.6609

Using RNRM++, Sim(*a*, *e*) in Fig 3 is higher than it is in Fig 4. Since in Fig 3, users *b* and *d* have a link, which means a closer relationship, but do not in Fig 4, using RNRM++, a closer partnership results in a larger similarity score, Sim(*a*, *e*). Compared to Sim(*a*, *e*) obtained using RNRM, we find that Sim(*a*, *e*) obtained using RNRM++ has a much higher similarity, because no matter whether users *b* and *d* have a link, the link will be set to be computed.

#### The generalized SimRank neighbor weighting scheme

As described above, we proposed an advanced initialization method and two novel neighbor ranging methods called RNRM and RNRM++. Similar to SimRank, they are both based on the idea of neighbor ranging. Here, we propose the generalized SimRank neighbor weighting method, which contains SimRank with the advanced initialization, RNRM and RNRM++. The detailed steps of this method are given as Algorithm 1.

### Algorithm 1: Generalized SimRank neighbor ranging algorithm

Input: a set of connection data, a set of parameters *K*, *γ*

Output: similarity matrix

1. assign the similarity of a pair of nodes *a*, *b*:

R0(a,b)={1if(a=b)1I(a)*I(b)if(a^b)0else

2. assign I(*v*) for each node *v*: I(*v*) = neighbors(*v*)

3. n: the number of nodes

4. for *k* = 1: *K*

5.    for *a* = 1 to n, do

6.     for *b* = 1 to n, do

7.       Calculate the similarity of *a* and *b*:

$$S_{k}(a,b)=\frac{\gamma }{\left|I(a)||I(b)\right|}\sum_{i=1}^{I(a)}\sum_{j=1}^{I(b)}\left[S_{k-1}(I_{i}(a),I_{j}(b))\times D^{*}(I_{i}(a),I_{j}(b))\right]$$

8. Return a similarity matrix

$$\begin{array}{rr} {}*D^{1}(I_{i}(a),I_{j}(b))= & \begin{array}{rr} 1 & \text{condition}\;1 \end{array}\\ D^{2}(I_{i}(a),I_{j}(b))= & \{\begin{array}{rr} 1 & \text{condition}\;2\\ 0 & \text{else} \end{array}\\ D^{3}(I_{i}(a),I_{j}(b))= & \{\begin{array}{rr} 1 & \text{condition}\;3\\ 0 & \text{else} \end{array} \end{array}$$

 condition 1:

  *a* = *b*

 condition 2:

  *I*_*i*_(*a*)∈*I*(*b*)∪*b* and *I*_*i*_(*b*)∈*I*(*a*)∪*a*

 condition 3:

  *I*_*i*_(*a*)∈*I*(*b*)∪*b* and *I*_*i*_(*b*)∈*I*(*a*)∪*a* and *I*_*i*_(*a*)∪*I*_*i*_(*b*)

In Algorithm 1, *γ* is a constant between 0 and 1, *I* (•) denotes the set containing node •’s neighbors, and D*(*I*_*i*_(*a*), *I*_*j*_(*b*)) contains values corresponding to three different conditions. For condition 1, it has a constant value of 1, indicating that the similarities of all pairs of nodes can be incorporated into the computation. Here, condition 1 stands for the SimRank. For condition 2, it represents the RNRM method, in which only if a pair of nodes are common friends of *a* and *b* can their similarity be considered in the computation. In condition 3, a limitation is added to condition 2 that only if the common friends themselves have a direct connection can they contribute to the similarity of users *a* and *b*.

This algorithm integrates the methods above and has all of their advantages. SimRank can be regarded as a special case in our scheme. The output of our scheme is a similarity matrix that includes the similarity of each pair of users in the community. Its entries are used as the weights of edges in future steps.

### Weighted fast Newman algorithm

The fast Newman algorithm is an agglomerative hierarchical clustering method. The algorithm starts with the state that each node is regarded as a separate community, and repeatedly joins two communities together in each step until only one community remains. The criterion of joining two communities is that the merging of these two communities yields the greatest increase (or smallest decrease) in the modularity *Q*. The decomposition with the largest *Q* value is selected as the final community structure. Thus, the fast Newman algorithm is actually a greedy algorithm to optimize the modularity *Q* of a given network.

For an unweighted network, we denote the increased value of the modularity *Q* by Δ*Q*_*st*_ when communities *s* and *t* are merged. Then, according to the definition of *Q*, it is easy to obtain

$$\Delta Q_{st}=\frac{1}{m}\sum_{i\in s,\;j\in t}\left(A_{ij}-\frac{k_{i}k_{j}}{2m}\right)$$ (11)

To extend the fast Newman algorithm to weighted networks, we only need to extend the definition of Δ*Q*. Similarly, we denote the increased value of the weight modularity *Q*^*w*^ by Δ*Q*^*w*^_*st*_ when communities *s* and *t* are merged. According to the definition of *Q*^w^,

$$\Delta Q_{st}^{w}=\frac{1}{m}\sum_{i\in s,\;j\in t}\left(w_{ij}-\frac{T_{i}T_{j}}{2m}\right)=2\left(e_{ij}-a_{i}a_{j}\right)$$ (12)

With this definition of Δ*Q*^*w*^, the normal fast Newman algorithm merging greedily maximizes the weighted modularity *Q*^*w*^.

### Communication-based modularity

In the process of detecting community structure, we have to measure the division. A standard measure is utilized to estimate how reasonable a decomposition of a given network is, so that the community discovery algorithms can be ranked. Various measures have been proposed in the literature, including the fraction of the nodes that are classified correctly [31], the Jaccard index [32] and the modularity proposed by Girvan and Newman [21], which is the most prevalent measure. The modularity function is used to measure the difference between the current community structure and the community structure in a random structure. These modularity measures are all based on the topology of the network. However, the topology cannot completely measure the inner social information contained in the network. For users who use a social network to obtain information and communicate with their friends, we believe the communication can represent this significant information and most of the users send messages to friends concentrated in their own societies, while fewer users send messages to users connected through other associations. Based on this assumption, we propose a new modularity measure named *Q*_*C*_, which represents that there should be as little exchange of communication as possible between communities in a social network. It is defined in Eq (13).

$$Q_{c}=\frac{N_{out}}{C_{n}^{2}}$$ (13)

where *N*_*out*_ denotes the number of communications between different communities, *n* is the number of communities in the final partition, $C_{n}^{2}$ denotes the total number of connections between any two communities, and *Q*_*C*_ is the average number of connections in different communities.

The communication mainly includes two parts: One is the micro-blog retweet, where a user retweets a micro-blog posted by another user, which can be regarded as a single communication. This is because in this retweet, the user will add his or her ideas and comments to the original post, which is an interflow with the original user. The other is communication using the symbol @. In a Sina micro-blog, if the symbol @ with a target user’s screenname after it appears in a message, the message will be sent to the target user directly. The target user can see this message in another section of the webpage after receiving a notification. A micro-blog without the symbol @ is posted to the entire audience, whereas by using @, a user usually aims the message at a particular friend. Naturally, the symbol @ represents a single communication. In other words, the number of communications is the sum of the numbers of communications in these two parts.

## Experimental results

### Datasets

We collected micro-blog data using an API provided by the Sina Micro-blog open platform for our experiments. In the process of obtaining data, we strictly complied with the terms of service for the micro-blog website. The crawler program started from some seed users and downloaded data according to the following relationships, using the principle of breadth-first traversal. Users who have few friends are more likely to be “zombie fans” and a user who has too many friends may represent an organization. Therefore, we chose four users as seed users randomly in the micro-blog network who have between 50 and 100 friends. As a result, four groups of datasets were captured, which contain the information of four seed users and the corresponding two-way following relationships that originate from them. Table 4 describes the four datasets (S1–S4 Files).

**Table 4:** Four datasets for the experiments.

Dataset	Number of users	Number of following relationships	Number of micro-blogs
Sina-Data1	5,000	17,044	618,271
Sina-Data2	8,734	84,311	1,476,810
Sina-Data3	5,649	40,284	939,194
Sina-Data4	6,502	28,909	872,743

**Fig 5:**
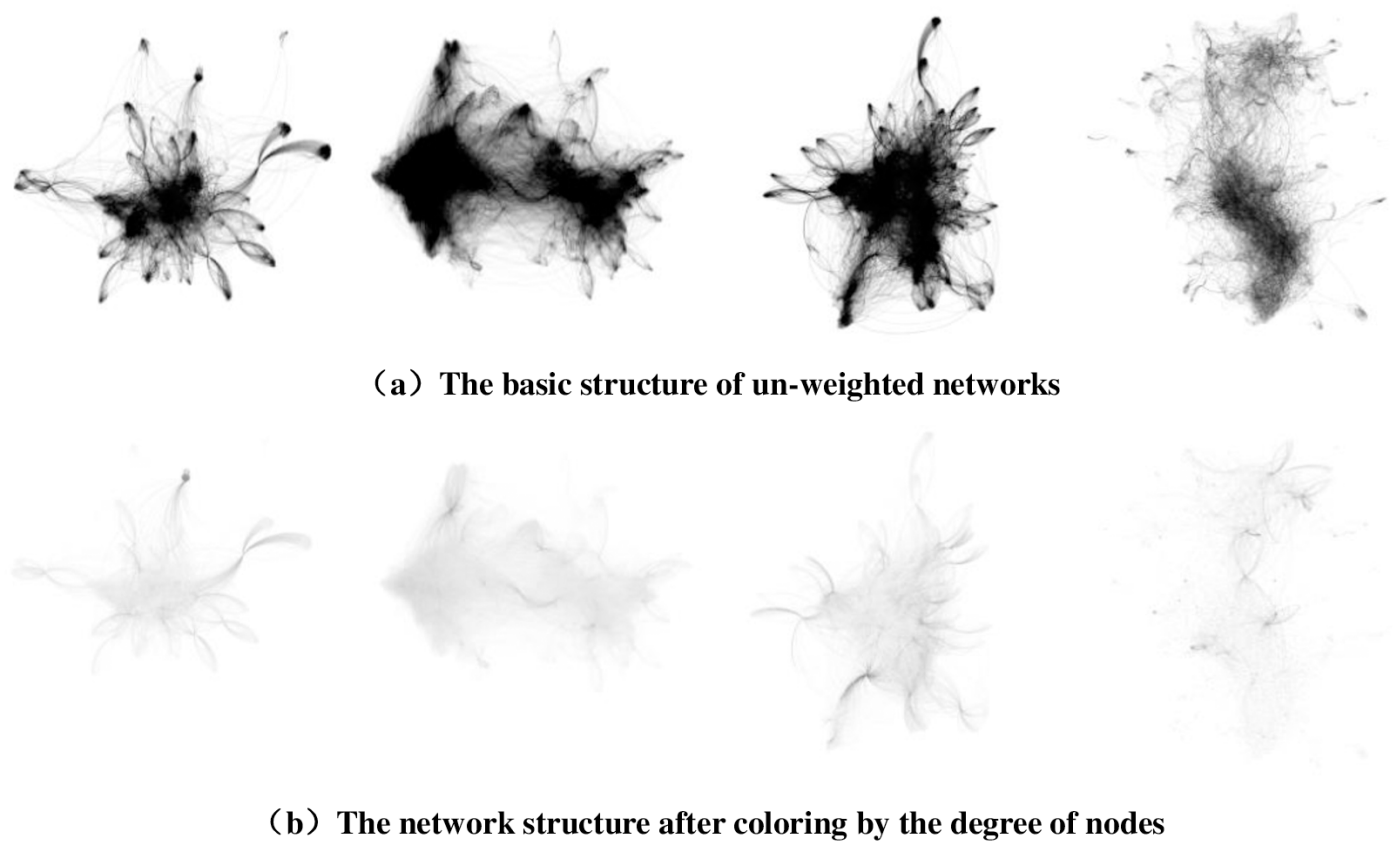
Micro-blog network structures of the four datasets.

**Fig 6:**
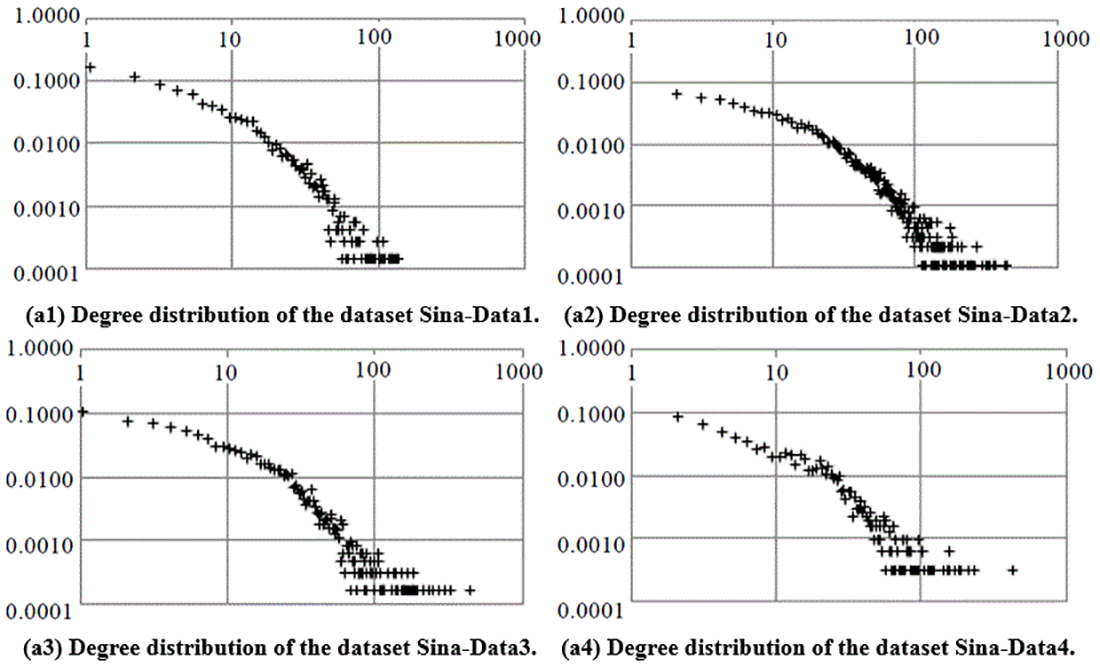
Degree distributions of micro-blog networks of the four datasets.

Fig 5 shows the micro-blog network structures of the four datasets. Fig 5(a) shows the basic structures of the un-weighted networks of datasets Sina-Data1 (S1 File), Sina-Data2 (S2 File), Sina-Data3 (S3 File) and Sina-Data4 (S4 File). It can be concluded from the figures that all four networks are connected graphs, whose nodes are connected with one another and there are no isolated nodes or node groups. In Fig 5(b), we color every network according to the degrees of nodes to show the degree distributions of these four groups of data (the higher the node degree is, the deeper the color is). We can see that a large number of nodes have a low degree in the micro-blog network and only a few have a high degree. Fig 6 depicts the detailed degree distributions of the micro-blog networks of the four datasets, which further reveals the distribution characteristics of these networks. Fig 6 indicates that the degree distributions of all four networks formed by the four corresponding groups of data roughly obey power-law distributions. Therefore, the four micro-blog networks are all scale-free networks [33].

### Compared algorithms

We proposed a generalized SimRank Neighbor Weighting scheme, including the SimRank method with advanced initialization, RNRM method and RNRM++ method. When these methods are used to extend the fast Newman algorithm for community discovery, the corresponding algorithms are called the modified fast Newman method with advanced SimRank (FNAS), modified fast Newman with RNRM (FNRNRM) and modified fast Newman with RNRM++ (FNRNRM++). We apply these algorithms to our four groups of data. The results are compared with those of the fast Newman algorithm [22], CNM [23], LC [12] and the Extended Link Clustering(ELC) [15] to verify the validity of our proposed algorithms. Here, the algorithms to be compared are briefly introduced.

The fast Newman algorithm. The fast Newman algorithm is a fast complex network community algorithm based on local search, whose optimization goal is to maximize the modularity evaluation function *Q*. The local search strategy for the candidate solution is to select and merge two existing network clusters. From the initial solution (each network cluster contains only one node), in each iteration, the algorithm performs a merge operation that maximizes the Δ*Q* value until only one network cluster is left in the network.

CNM. Based on the fast Newsman algorithm, CNM is a kind of algorithm that discovers the community structure in the network by introducing the modularity of the incremental matrix and the heap structure.

LC. LC takes edges as the research object. By calculating the Jaccard distance between each pair of edges, a similarity matrix that measures the degree of similarity between edges is obtained. The row vectors in the similarity matrix are regarded as points in the Euclidean space, and these points are clustered by the method of hierarchical clustering. Finally, the tree division spectrum obtained by hierarchical clustering is divided according to the division density, and the optimal classification level and the results of community division are obtained.

ELC. This is a community discovery algorithm for extended edge clustering. After calculating the similarity matrix between edges according to the extended edge similarity, the improved similarity matrix is clustered using the hierarchical clustering method, which is similar to the LC algorithm.

### Analysis of results

This section first compares the convergence rates of different edge weighting methods and presents the user’s similarity matrices on four groups of datasets that are calculated by different edge weighting methods. Second, we compare the advantages and disadvantages between our algorithms and other algorithms from the perspective of community detection. Our new modularity *Q*_*C*_ method and classical modularity *Q* method are used as evaluation indices.

#### Convergence rates of algorithms

To measure the rates of convergence of the algorithms, we calculated the difference of similarity matrices in each iteration. This helps indicate the convergence rate in Eq (14),

$$D_{k}=\sum_{i=1}^{N}\sum_{j=1}^{N}(S_{ij}^{k}-S_{ij}^{k-1}{)}^{2}$$ (14)

where $S_{ij}^{k}$ denotes the similarity of user *i* and user *j* in the *K*th iteration, *N* is the number of nodes in this network, and *D*_*k*_ is the difference in the similarity between iteration *k* and iteration *k*-1 of the whole network.

**Fig 7:**
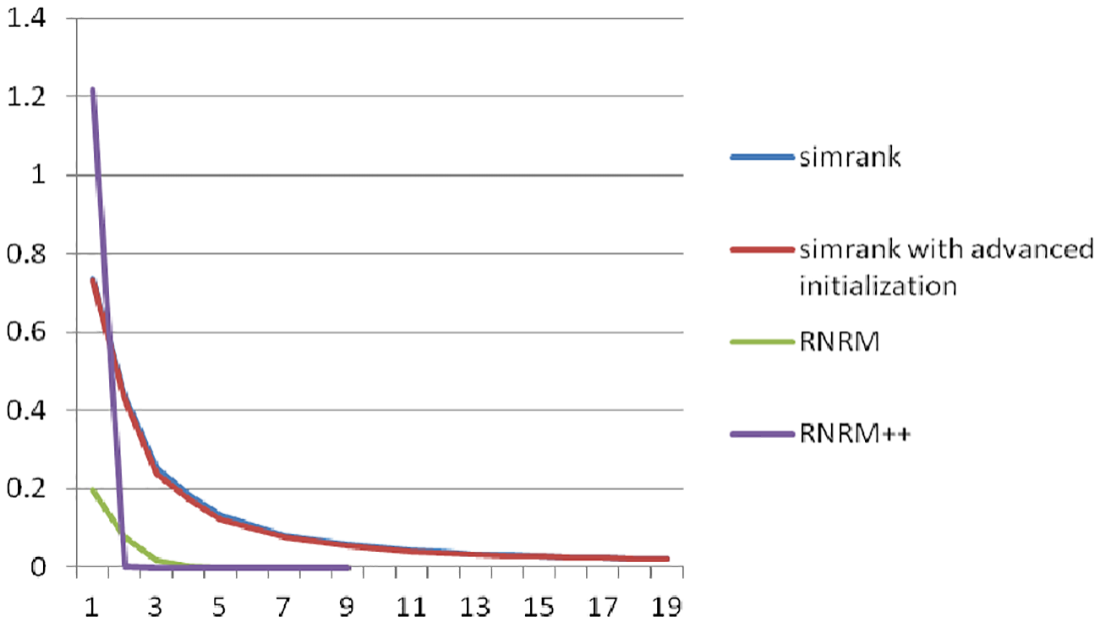
The convergence rates of algorithms using Fig 3.

Fig 7 shows the convergence rates using the network in Fig 3 with the original SimRank, SimRank with advanced initialization, RNRM and RNRM++. Regardless of the value in the first iteration, the rate of convergence with RNRM++ is significantly faster than with the other methods. RNRM takes second place and the original SimRank and SimRank with advanced initialization, which share a basic coincidence line, have the slowest convergence rates. Although this is only a relatively small example, the line graph significantly proves that our algorithm converges faster. This would be particularly relevant for a social network with a large number of nodes.

**Fig 8:**
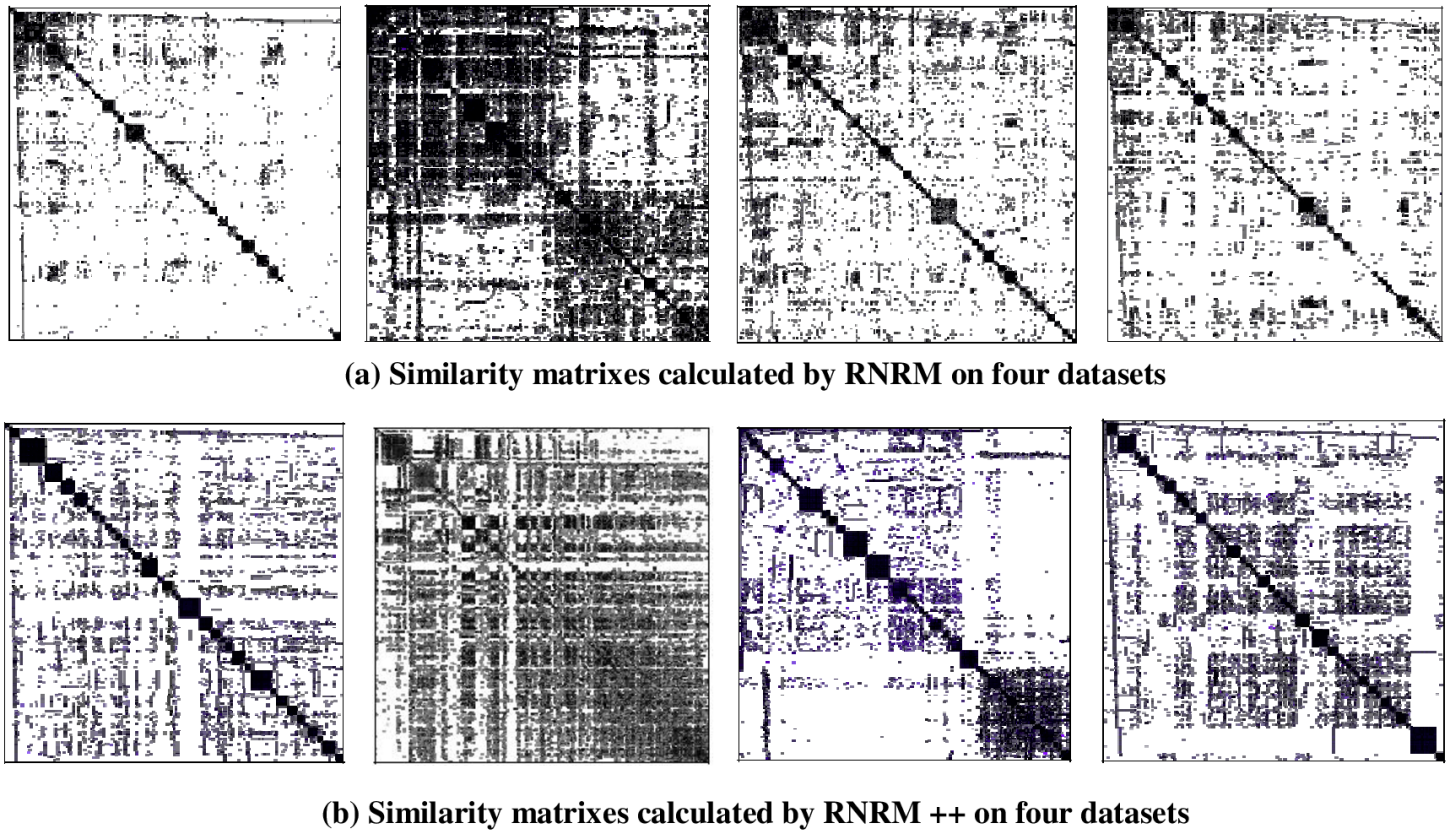
Similarity matrices calculated by RNRM and RNRM ++ using two different edge weighting methods.

To show the similarity between micro-blog users in each dataset more clearly, we represent the similarity matrices calculated by RNRM and RNRM ++ in two edge weighting methods in graphic form. Fig 8 shows the results on four datasets. In the similarity matrix graph, the deeper the color of a point is, the higher the similarity value of the corresponding user pair.

#### User community discovery in the Sina Micro-blog networks

To evaluate the effectiveness of the proposed edge weighting method, we first analyze dataset Sina-Data1 with information on 5000 users from weibo.com. The dataset contains each user’s following list and communication information. Then, we build a network of following relationships: two users may a connection if one user follows the other. After this, we obtain a large network with some small bipartite graphs, and then we select the largest connected bipartite graph, with 457 users and more than 3000 edges, for the following experiment.

**Fig 9:**
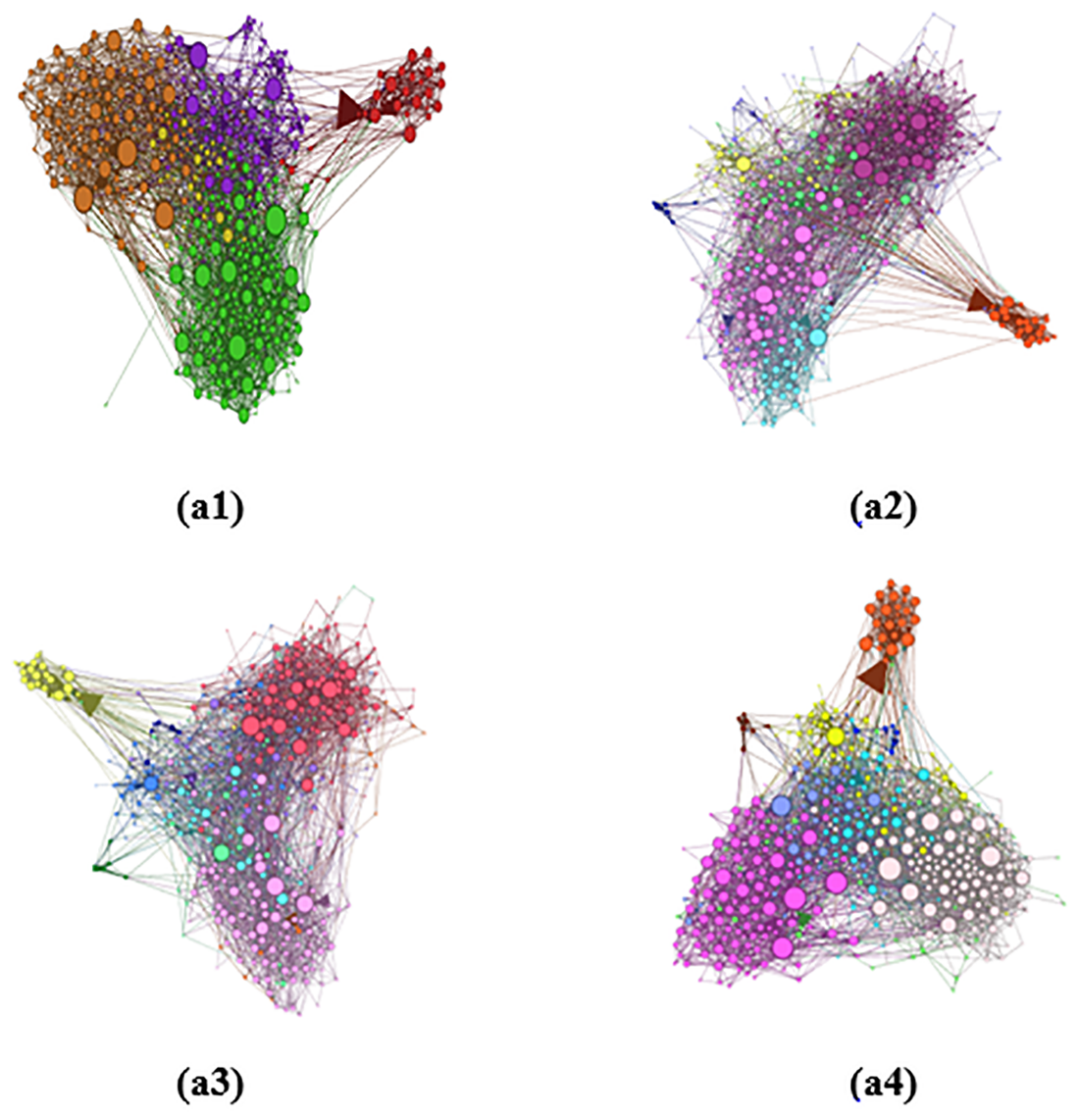
Illustrations of the detection results with different algorithms for dataset Sina-Data1. (a1) the fast Newman algorithm, (a2) FNAS, (a3) FNRNRM, (a4) FNRNRM++.

The Generalized SimRank Neighbor Ranging Scheme with different conditions is implemented for comparison. The proposed weighting scheme and the Generalized SimRank Neighbor Ranging Scheme are combined with the weighted fast Newman algorithm for application to real-world networks. To better understand the effects of the weighting schemes, the original fast Newman algorithm was also performed. Fig 9 shows the detection results with the different algorithms. Each community is shaded with a different color. Since there are too many links and nodes in the network, we cannot distinguish the results clearly.

Since we cannot easily evaluate the results in Fig 9, we use a new measure, *Q*_*C*_, in Eq (13) to weight the different methods. As mentioned before, *Q*_*c*_ can measure the practical significance of the network. In Table 5, FNRNRM and FNRNRM++ achieve almost the same scores, which are much lower than those of Fast Newman and FNAS. This means that after using FNRNRM and FNRNRM++, users’ communications will be concentrated within their own community. This accords with our real-life behavior, that we usually chat with friends in our own circle, and shows that our method is suitable for real networks.

**Table 5:** Experimental results of modularity *Q*_*C*_ for dataset Sina-Data1.

Algorithm	Number of communities	*Q*_*c*_
the fast Newman algorithm	7	31.28
FNAS	8	25.5
FNRNRM	10	16.73
FNRNRM++	9	19.72

Through these experiments, we show that the proposed weighting scheme is able to improve the performance of the original fast Newman algorithm significantly and is superior to FNAS in both quality of results and calculation speed.

We further verify the effectiveness of our algorithms in community detection. Table 6 shows the modularity *Q* values of communities discovered by our algorithms and the compared algorithms on four datasets. First, the CNM algorithm has the largest modularity for all four datasets, with values of 0.315, 0.196, 0.324, and 0.362. According to [21], a good community detection algorithm should have a modularity value between 0.3 and 0.7. For all datasets except Sina-Data2, the modularity of CNM falls into this range. However, the modularity is enhanced for CNM, which finds only non-overlapping communities; this results in the detection of fewer communities compared to the other algorithms. LC has the lowest modularity for four datasets, with values of 0.112, 0.031, 0.049, and 0.098. This is because the algorithm clusters according to edges and generates the largest number of communities among all compared methods, of which most are small and isolated communities (e.g., a community formed by two nodes and one edge between). ELC improves the computation of edge similarity over LC, giving a slight enhancement of modularity. Lastly, our proposed FNAS, FNRNRM and FNRNRM++ algorithms achieve better modularity values than the Fast Newman algorithms by considering node-node similarity and applying it to the weighted Fast Newman algorithm, and thus have better performance on community division. Specifically, the modularity values of FNRNRM (resp., FNRNRM++) on the four datasets are 0.137 (resp., 0.141), 0.152 (resp., 0.154), 0.211 (resp., 0.209), and 0.230 (resp., 0.248). Both algorithms achieve better modularity values than FNAS (with FNRNRM++ outperforms a little bit) due to their more reasonable strategies of node similarity computation. Overall, the proposed algorithms in our paper with the weighted edge method applied to the extended Fast Newman algorithm have notable effects on community division.

**Table 6:** Modularity *Q* values in our algorithms and compared algorithms.

Dataset	Modularity *Q*						
	the fast Newman algorithm Newman	CNM	LC	ELC	FNAS	FNRNRM	FNRNRM++
Sina-Data1	0.128	0.315	0.112	0.121	0.129	0.137	0.141
Sina-Data2	0.094	0.196	0.031	0.047	0.125	0.152	0.154
Sina-Data3	0.151	0.324	0.049	0.093	0.164	0.211	0.209
Sina-Data4	0.163	0.362	0.098	0.126	0.169	0.230	0.248

## Discussion

In this paper, a detailed discussion on the problem of the local and global weighting balance was first presented. The Generalized SimRank Neighbor Ranging Method was proposed as a novel edge weighting method, which contains multiple novel neighbor ranging methods, and SimRank, which is one of its special cases, was demonstrated to meet the requirements of balance quite well. Then, the fast Newman algorithm was extended to weighted networks. Combined with the edge weighting techniques, the extended algorithm enhanced the performance of the original algorithm significantly and achieved a faster convergence rate. Experimental results on real-world networks have verified the effectiveness of the new weighting scheme.

This study also suggests some directions for further research. First, more candidate techniques can be developed for adaption to different kinds of networks. Second, the proposed method can be applied to a larger real-world network or even be extended to address networks with more attributes. Third, it would be interesting to apply the new weighting scheme to other community discovery algorithms.

## Appendix

In the appendix, we mainly elaborate on the datasets that were used in our experiments. First, we would like to state that the approach to collecting data strictly obeyed the terms of service from the Sina Micro-Blog website. There are two ways to obtain the data of a micro-blog. One is to extract the data using software such as “Spider Duck”. The other is to call the public API provided by the Sina Micro-Blog open platform to acquire data. We chose the latter because of its customization. Take the dataset Sina-Data1as an example to explain the procedure of obtaining it. The steps are as follows: First, choose a seed user randomly, and then grab his friend list and fan list using the API. Next, both the “friend list” and the “fan list” are considered the new “seed users”. Grab the “friend list” and “friend list” of each seed user; thus, the grab process is conducted recursively. In the pre-grab stage, we collect 8619 users and 23195 following relations. The data can’t be used directly because these relations are too sparse; many users only have a one-way connection with the “core user”, in other words, they only follow the “core user” and don’t have following relations with other users. Therefore, we conduct selection of the following relations and filter out the one-way connections. The resulting dataset contains 5000 users, who have 17044 two-way connections with other users in total.

## Supporting information

S1 File (RAR)

S2 File (RAR)

S3 File (RAR)

S4 File (RAR)
