# Correction: Micro-blog user community discovery using generalized SimRank edge weighting method

**DOI:** 10.1371/journal.pone.0198879

**Published:** 2018-06-06

**Authors:** The PLOS ONE Staff

The second author's name is incorrect. The correct name is: Xun Liang. The correct citation is: Qi J, Liang X, Zhou X, Li Z, Liu Y, Cheng H (2018) Micro-blog user community discovery using generalized SimRank edge weighting method. PLoS ONE 13(5): e0196447. https://doi.org/10.1371/journal.pone.0196447. The publisher apologizes for the error.
